# Optimal experiment design for model selection in biochemical networks

**DOI:** 10.1186/1752-0509-8-20

**Published:** 2014-02-20

**Authors:** Joep Vanlier, Christian A Tiemann, Peter AJ Hilbers, Natal AW van Riel

**Affiliations:** 1 Eindhoven University of Technology, Department of Biomedical Engineering, PO Box 513, Eindhoven, 5600 MB, The Netherlands; 2 Netherlands Consortium for Systems Biology, University of Amsterdam, Amsterdam, 1098 XH, The Netherlands

**Keywords:** Model selection, Inference, Bayes factor, Uncertainty

## Abstract

**Background:**

Mathematical modeling is often used to formalize hypotheses on how a biochemical network operates by discriminating between competing models. Bayesian model selection offers a way to determine the amount of evidence that data provides to support one model over the other while favoring simple models. In practice, the amount of experimental data is often insufficient to make a clear distinction between competing models. Often one would like to perform a new experiment which would discriminate between competing hypotheses.

**Results:**

We developed a novel method to perform Optimal Experiment Design to predict which experiments would most effectively allow model selection. A Bayesian approach is applied to infer model parameter distributions. These distributions are sampled and used to simulate from multivariate predictive densities. The method is based on a *k*-Nearest Neighbor estimate of the Jensen Shannon divergence between the multivariate predictive densities of competing models.

**Conclusions:**

We show that the method successfully uses predictive differences to enable model selection by applying it to several test cases. Because the design criterion is based on predictive distributions, which can be computed for a wide range of model quantities, the approach is very flexible. The method reveals specific combinations of experiments which improve discriminability even in cases where data is scarce. The proposed approach can be used in conjunction with existing Bayesian methodologies where (approximate) posteriors have been determined, making use of relations that exist within the inferred posteriors.

## Background

**Figure 1:**
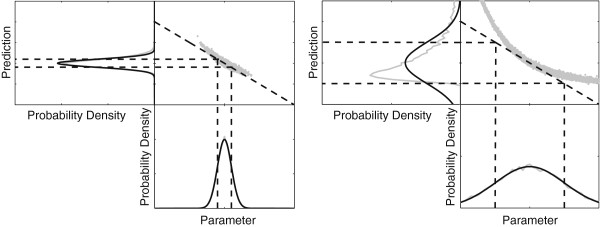
**Limitations of estimating prediction variances using linearization.** In this figure, the gray dots indicate model predictions corresponding to simulations of the nonlinear model. On the prediction side, the black distribution is based on linearly projecting the parameter uncertainties onto the predictions, while the gray distribution is based on the non-linear model. Shown on the left is the case with small uncertainty, where the linear parameter sensitivities provide an adequate description for projecting the parameter uncertainty onto the predictions as can be seen from the overlapping black and gray lines. On the right, the case with large parameter uncertainty, where the non-linearity of the model results in a poor estimate of the predictive distribution when it is estimated via linear projection i.e. the black and gray lines do not overlap.

Developing computational models of biochemical networks is complicated by the complexity of their interaction mechanisms [1-8]. Typically, hypotheses on how the system operates are formalized in the form of computational models [9-12]. These models are subsequently calibrated to experimental data using inferential techniques [13-19]. Despite the steady increase in data availability originating from new quantitative experimental techniques, the modeler is often faced with the problem that several different model topologies can describe the measured data to an acceptable degree [20-22]. The uncertainty associated with the predictions hinders the investigator when trying to make a clear distinction between competing models. In such cases, additional data is required. Optimal Experiment Design (OED) methods can be used to determine which experiments would be most useful [23]. These methods typically involve specifying an optimality criterion or design aim and finding the experiment that most effectively attains this goal while considering the current parameter uncertainty. Existing methods of OED for model selection are usually based on assuming an uncertainty distribution around best parameter estimates [24,25] or model linearization [26]. Due to the non-linearity of the model and the non-Gaussian shape of the parameter distribution, these methods are rarely appropriate for Systems Biology models [27] (See Figure 1 for an example of the effect of model linearization, and how it can skew predictive distributions in cases of large parameter uncertainty). In this work, we employ a Bayesian approach using the Posterior Predictive Distribution (PPD) which directly reflects the prediction uncertainty and accounts for both model non-linearity and non-Gaussianity of the parameter distribution. PPDs are defined as distributions of new observations conditioned on the observed data. Samples from the PPD can be obtained by drawing from the posterior parameter probability distribution and simulating predictions for each parameter set. By simulating a sample from the PPDs for all experimentally accessible moieties and fluxes, differences between models can be explored [28].

Previously, predictive distributions have been used to perform experiment design targeted at reducing the uncertainty of specific predictions [29-31]. In the field of machine learning, optimal experiment design based on information-theoretic considerations is typically referred to as active learning [32]. In the neurosciences, the Bayesian inversion and selection of nonlinear states space models is known as dynamic causal modelling (DCM). Although DCM is dominated by variational (approximate) Bayesian model inversion - the basic problems and ensuing model selection issues are identical to the issues considered in this work. In DCM, the issue of optimising experimental design focuses on the Laplace-Chernoff risk for model selection and its relationship with classical design optimality criteria. Daunizeau et al. (2011) consider the problem of detecting feedback connections in neuronal networks and how this depends upon the duration of design stimulation [33]. We will consider a similar problem in biochemical networks - in terms of identifying molecular interactions and when to sample data. We present a method to use samples from simulated predictive distributions for selecting experiments useful for model selection. Considering the increased use of Bayesian inference in the field [14,34-39], this approach is particularly timely since it enables investigators to extract additional information from their inferences.

**Figure 2:**
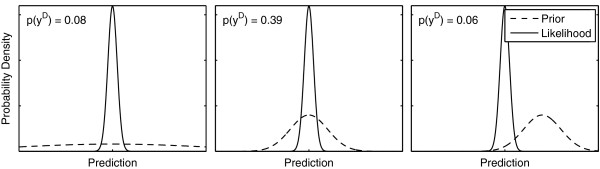
**Effect of model complexity on marginal likelihood.** Three different illustrative examples of integrated likelihoods. Left: Integrated likelihood under wide priors. The mismatch of the prior with respect to the high likelihood region results in low weights for the high likelihood region and therefore low model evidence. This situation is comparable to a case where the model contains too many parameters. A surplus of model parameters leads to a larger parameter space and therefore lower weights in high likelihood region. Middle: A good match between prior and likelihood. Right: A model that does not have sufficient freedom to describe the data very well.

In a Bayesian setting, model selection is typically based on the Bayes factor, which measures the amount of evidence the data provides for one model over another [40,41]. For every pair of models, a Bayes factor can be computed, defined as the ratio of their integrated likelihoods. One advantage of the Bayes factor is that it automatically penalizes unnecessary model complexity in light of the experimental data. It therefore reduces the risk of unwarranted model rejections. This penalization occurs because more parameters or unnecessarily wide priors lead to a lower weighting of the high likelihood region. This is illustrated in Figure 2.

**Figure 3:**
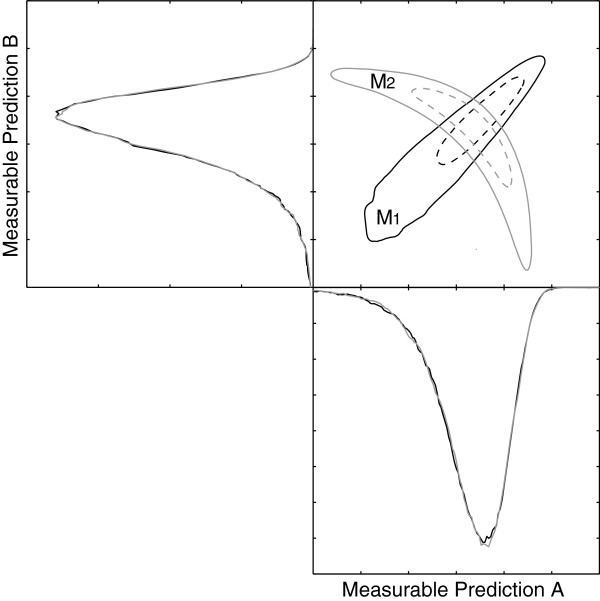
**Different models can imply different inter-prediction relations.** An illustrative example of how different models can imply different relations between predictions. On the top right are the 67% (dashed) and 95% (solid) probability contours of the joint probability density functions of two predictions obtained with model *M*_1_ and *M*_2_, while the other two panels show the distribution of that specific prediction. Note how measuring one of the two predictions would yield no additional discriminatory power while measuring both predictions would.

What the Bayesian selection methodology does not provide, however, is a means to determine which experiment would optimally increase the separation between models. Determining which measurements to perform in order to optimally increase the Bayes factor in favor of the correct model is a difficult task. We propose a method which allows ranking combinations of new experiments according to their efficacy at increasing the Bayes factors which point to the correct model. Predictions whose distributions do not overlap between competing models are good measurement candidates [42,43]. Often distributions for a single prediction show a large degree of overlap, hampering a decisive outcome. Fortunately, PPDs also contain information on how model predictions are related to each other. The relations between the different prediction uncertainties depend on both the data and the model. Differences in these inter-prediction relations between competing models can be probed and used (see Figure 3). We quantify these differences in predictive distributions by means of the Jensen Shannon divergence (JSD) [44].

There are many design parameters that one could optimize. In this paper, we focus on a simple example: namely, which system variable should be measured and at which time point. We argue that by measuring those time points at which the models show the largest difference in their predictive distributions, large improvements in the Bayes factors can be obtained. By applying the methodology on an analytically tractable model, we show that the JSD is nearly monotonically related to the predicted *change* in Bayes factor. Subsequently, the Jensen Shannon divergence is computed between predictions of a non-linear biochemical network. Since each model implies different relations between the predictive distributions, certain combinations of predictions lead to more discriminability than others. The method serves as a good predictor for effective experiments when compared to the obtained Bayes factors after the measurements have been performed. The approach can be used to design multiple experiments simultaneously, revealing benefits that arise from combinations of experiments.

## Methods

Consider biochemical networks that can be modeled using a system of ordinary differential equations. These models comprise of equations $f(\vec{x}(t),\vec{u}(t),\vec{p})$ which contain parameters $\vec{p}$ (constant in time), inputs $\vec{u}(t)$ and state variables $\vec{x}(t)$. Given a set of parameters, inputs, and initial conditions $\vec{x}(0)$, these equations can be simulated. Measurements $\vec{y}(t)=g(\vec{x}(t),\vec{q},\vec{r})$ are performed on a subset and/or a combination of the total number of state variables in the model. Measurements are hampered by measurement noise $\vec{\varepsilon }$, while many techniques used in biology necessitate the use of scaling and offset parameters $\vec{r}$[45]. The vector $\vec{\theta }$, defined as $\vec{\theta }=\{\vec{p},\vec{r},{\vec{x}}_{0}\}$, lists all the required quantities to simulate the model. The parameters $\vec{q}$ determine the experimental design and could include differences in when various responses are measured or the mapping from hidden model states $\vec{x}$ to observed responses $\vec{y}$. We will refer to these as ‘design parameters’ that are, crucially, distinguished from model parameters $\vec{\theta }$. Design parameters are under experimental control and determine the experimental design. In what follows, we try to optimise the discriminability of models in terms of Bayesian model comparison by optimizing an objective function with respect to $\vec{q}$. In the examples, we will consider $\vec{q}$ as the timing of extra observations.

To perform inference and experiment design, an error model is required. Considering *R* time series of length *N*_1_, *N*_2_ … *N*_*R*_, hampered by independent noise, one obtains the equation:

(1) $$p\;\left(y^{D}|\;\vec{\theta },M_{i}\right)=\overset{R}{\underset{k=1}{\prod }}\overset{N_{k}}{\underset{j=1}{\prod }}p\;\left(y_{k}^{D}\;\left(t_{j}\right)\;,\vec{\theta },M_{i}\right)$$

where *M*_*i*_ indicates a model and *y*^*D*^ the observed data. The parameters are given by $\vec{\theta }$, while $y_{k}^{D}(t_{j})$ indicates the value of a data point of state variable *k* at time *j*, respectively.

### Predictive Distributions

Posterior Predictive Distributions (PPDs) are defined as distributions of new observations conditioned on the observed data. They correspond to the predicted distribution of future experiments, considering the current model assumptions and uncertainty. To obtain a sample from these predictive distributions, we propagate the uncertainty from the data to the predictions. By specifying prior distributions on the parameters and applying Bayes rule, it is possible to define a posterior distribution over the parameters. The posterior parameter probability distribution $p\;(\vec{\theta }|\;y^{D})$ is given by normalizing the likelihood multiplied with the prior to a unit area:

(2) $$p\;\left(\vec{\theta }|\;y^{D}\right)=\frac{p\;\left(y^{D}|\;\vec{\theta }\right)\;p\;\left(\vec{\theta }\right)}{p\;\left(y^{D}\right)}=\frac{p\;\left(y^{D}|\;\vec{\theta }\right)\;p\;\left(\vec{\theta }\right)}{\int p\;\left(y^{D}|\;\vec{\theta }\right)\;p\;\left(\vec{\theta }\right)\;d\vec{\theta }}$$

where $p(y^{D}|\;\vec{\theta })$ is the distribution of the observed data given parameters $\vec{\theta }$. The parameter prior distributions $p(\vec{\theta })$ typically reflect the prior uncertainty associated with the parameters. To sample from the posterior parameter distribution, one needs to verify that the posterior distribution is proper. This can be checked by profiling the different parameters and determining whether the likelihood times the prior does not flatten out [28,46]. After checking whether the posterior distribution of parameters is proper, a sample from this distribution can be obtained using Markov Chain Monte Carlo (MCMC) [22,28]. MCMC can generate samples from probability distributions whose probability densities are known up to a normalizing factor [47] (see Additional file 1: section S1). A sample of the posterior parameter distribution reflects the uncertainty associated with the parameter values and can subsequently be used to simulate different predictions. The predictive distribution can now be sampled by simulating the model for each of the samples in the posterior parameter distribution and adding noise generated by the associated error model. The latter is required as future observations will also be affected by noise.

### Model selection

In a Bayesian setting, model selection is often performed using the Bayes factor [40,48,49]. This pivotal quantity in Bayesian model selection expresses the change of relative belief in both models after observing experimental data. By applying Bayes rule to the problem of assigning model probabilities, one obtains:

(3) $$p\;\left(M|\;y^{D}\right)=\frac{p\;\left(y^{D}|\;M\right)\;p(M)}{p\;\left(y^{D}\right)}$$

where *P*(*M*|*y*^*D*^) represents the probability of model *M* given observed data *y*^*D*^, while *P*(*M*) and *P*(*y*^*D*^) are the prior probabilities of the model and data, respectively. Rather than explicitly computing the model probability, one usually considers ratios of model probabilities, allowing direct comparison between different models. As the prior model probability can be specified *a priori* (equal if no preference is given), the only quantity that still requires evaluation is *P*(*y*^*D*^| *M*), which can be obtained by integrating the likelihood function over the parameters:

(4) $$p\;\left(y^{D}|\;M\right)=\;\int \;p\;\left(y^{D}|\;M,{\vec{\theta }}_{M}\right)\;p\;\left({\vec{\theta }}_{M}|\;M\right)\;d{\vec{\theta }}_{M}.$$

The Bayes factor is actually the ratio of these integrated (also named marginal or marginalized) likelihoods and is defined as:

(5) $$\;B_{12}=\frac{p\;\left(y^{D}|\;M_{1}\;\right)}{p\;\left(y^{D}|\;M_{2}\;\right)}=\frac{\int \;p\;\left(\;y^{D}|\;M_{1},{\vec{\theta }}_{M_{1}}\;\right)\;p\;\left(\;{\vec{\theta }}_{M_{1}}|\;M_{1}\;\right)\;d{\vec{\theta }}_{M_{1}}}{\int \;p\;\left(\;y^{D}|\;M_{2},{\vec{\theta }}_{M_{2}}\;\right)\;p\;\left(\;{\vec{\theta }}_{M_{2}}|\;M_{2}\;\right)\;d{\vec{\theta }}_{M_{2}}}$$

where *M*_1_ and *M*_2_ refer to the different models under consideration. Unnecessarily complex models are implicitly penalized due to the fact that these result in a lower weighting of the high likelihood region, which results in a lower value for the integrated likelihood. This is illustrated in Figure 2. This means that maximizing the model evidence corresponds to maintaining an accurate explanation for the data while minimizing complexity [50].

Bounds can be defined where the Bayes factor value becomes decisive for one model over the other. Typically, a ratio of 100:1 is considered decisive in terms of model selection [40,51]. In dynamic causal modelling, variational methods predominate, usually under the Laplace assumption. This assumes that the posterior density has a Gaussian shape, which greatly simplifies both the integration problems and numerics. Note that assuming a Gaussian posterior over the parameters does not necessarily mean that the posterior predictive distribution over the data is Gaussian (see Figure 1). Computing the required marginal likelihoods is challenging for non-linear problems where such asymptotic approximations to the posterior distribution are not appropriate. Here one must resort to more advanced methods such as thermodynamic integration (see Additional file 1: section S2) [52] or annealed importance sampling [40]. Though the Bayes factor is a useful method of model selection, determining what to measure in order to improve the Bayes factor in favor of the correct model is a non-trivial problem. As such, it provides a means to perform model selection, but not the optimal selection of data features.

### Experiment design

The approach is based on selecting measurements which provide the largest discriminatory power between competing models in terms of their predictive distributions. The design parameter in the proposed methodology corresponds to the choice and timing of a new observation. In other words, we want to determine which observable should be measured when in order to maximize the divergence between the posterior predictive densities, thereby maximally informing our model comparison. This divergence is quantified by means of the Jensen Shannon divergence (JSD) as it provides a measure of the dissimilarity between the probability density functions of competing models. The JSD is defined as the averaged Kullback Leibler divergence *D*_*KL*_ between probability distributions and their mixture:

(6) $$\begin{array}{c} \;D_{\text{JS}}=\overset{K}{\underset{i=1}{\sum }}p\;\left(M_{i}\right)\;D_{\text{KL}}\;\left(p\;\left(y|\;M_{i}\right)\;,\;\overset{K}{\underset{i=1}{\sum }}\;p\;\left(M_{i}\right)\;p\;\left(y|\;M_{i}\right)\right). \end{array}$$

Here *K* represents the number of probability densities, *p*(*M*_*i*_) the (prior) probability of model *M*_*i*_ and *p*(*y*| *M*_*i*_) the Posterior Predictive Distribution. Additionally, this metric is monotonically related to an upper and lower bound of the classification error rate in clustering problems [33,53] and is bounded between 0 and 1. In the case where the model that generated the data is in the set of competing models, it is analogous to the mutual information between a new measurement (or sample) coming from a mixture of the candidate models and a model classifier (see Additional file 1: section S3). In this study, we opted for comparing models in a pairwise fashion (*K*=2). This allows us to determine which models are distinguished by a new experiment. Mutual information has been considered before in the context of experimental design for constraining predictions or parameters of interest [29], but not in the setting of model selection. Though appealing for its properties, estimating the Jensen Shannon divergence for one or more experiments requires integration over the predictive densities, since:

(7) $$D_{\text{KL}}(P,Q)=\int p(x)\;\log_{2}\;\left(\frac{p(x)}{q(x)}\right)\;\mathrm{dx.}$$

Here *P* and *Q* are random variables with *p* and *q* their associated densities. Considering that only a sample of the PPDs is available, it is required to obtain a density estimate suitable for integration. Density estimation can be approached in two ways: by Kernel Density Estimation (KDE), or by k-Nearest Neighbor (kNN) density estimation. In Kernel Density Estimation (KDE), an estimate of the density is made by centering normalized kernels on each sample and computing weighted averages. This results in a density estimate with which computations can be performed. The kernels typically contain a bandwidth parameter which is estimated by means of cross validation [54,55].

For well behaved low dimensional distributions, KDE often performs well. Considering the strongly non-linear nature of both the parameter and predictive distributions, a Gaussian kernel with constant covariance is not appropriate. As the dimensionality and non-uniformity of the problem increases, more and more weights in the KDE become small and estimation accuracy is negatively affected [56]. Additionally, choosing an appropriate bandwidth by means of cross-validation is a computationally expensive procedure to perform for each experimental candidate.

With *k*-Nearest Neighbor (kNN) density estimation, density is estimated by computing the volume required to include the *k* nearest neighbors of the current sample [55-57]:

(8) $$p\;\left(\vec{\theta }\right)=\frac{1}{N}\frac{k}{\;\rho_{k}{\left(\vec{\theta }\right)}^{d}v_{d}}$$

In this equation $\rho_{k}(\vec{\theta })$ represents the distance to the *k*^*t* *h*^ nearest neighbor, *d* the number of dimensions and *v*_*d*_ the volume of the unit ball in $R^{d}$. Furthermore, *N* denotes the number of included samples and *v*_*d*_ is given by:

(9) $$v_{d}=\frac{\pi^{d/2}}{\Gamma (d/2+1)}$$

where *Γ* corresponds to the Gamma function. The advantage of using the kNN estimate is that this estimator adapts to the local sampling density, adjusting its volume where sampling is sparse. Note, however, that, similar to the KDE, this estimator also suffers from a loss of accuracy when estimating high dimensional densities. Fortunately, the number of experiments designed simultaneously, and therefore the dimensionality of the density, is typically low. Consider ${\vec{y}}_{j}^{M_{i}}$, a vector of predictions simulated with model *M*_*i*_ and parameter set ${\vec{\theta }}_{j}$, where each element of the vector corresponds to a different model prediction. A model prediction is defined as a quantity which can be computed by supplying model *M*_*i*_ with parameter set ${\vec{\theta }}_{j}$ (e.g., a predicted value at a certain time point, a difference between predictions or an area under some predicted curve). For OED purposes, these should be quantities that could potentially be measured. The set of these predicted values coming from model *M*_*i*_ shall be referred to as $\Omega_{M_{i}}$. Inserting these quantities, the kNN estimate of the JSD becomes:

(10) $$\;D_{\text{js}}=\frac{1}{2N_{M_{1}}}\overset{N_{M_{1}}}{\underset{i=1}{\sum }}\log_{2}\;\left(Q_{1,2}(i)\right)+\frac{1}{2N_{M_{2}}}\overset{N_{M_{2}}}{\underset{i=1}{\sum }}\log_{2}\;\left(Q_{2,1}(i)\right)$$

with *Q*_*a*,*b*_ given by

(11) $$\begin{array}{c} \;Q_{a,b}(i)=\frac{2N_{M_{b}}r_{k}{\;\left({\vec{y}}_{i}^{M_{a}},\;\Omega^{M_{b}}\right)}^{d}}{N_{M_{b}}r_{k}\;{\left({\vec{y}}_{i}^{M_{a}},\;\Omega^{M_{b}}\;\right)}^{d}\;+\;(N_{M_{a}}\;-\;1)\;r_{k}\;{\left({\vec{y}}_{i}^{M_{a}},{\;\Omega }^{M_{a}}\setminus {\vec{y}}_{i}^{M_{a}}\;\right)}^{d}} \end{array}$$

**Figure 4:**
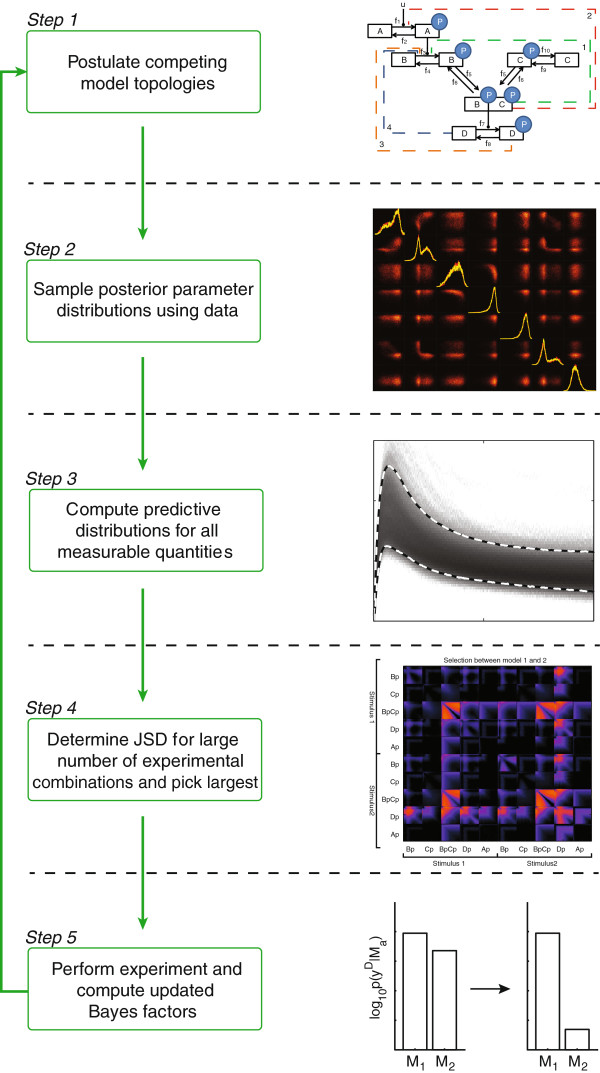
**Different steps of the proposed methodology.** Different steps of the proposed methodology. Step 1: Different model topologies have to be formulated into a form with which simulations can be obtained. Step 2: Posterior distributions of parameters have to be sampled. In the panel, the joint posterior probability distributions of a few model parameters are depicted, with their marginals on the diagonal. Step 3: Use sample from the posterior parameter distribution to make predictions of quantities that can be measured (a sample of the Posterior Predictive Distribution). Step 4: Compute JSD between the predictive distribution of different models. Step 5: Perform the optimal experiment and compute the new Bayes factors. Optionally repeat until the hypotheses are sufficiently refined.

Here *d* denotes the number of elements in ${\vec{y}}_{j}^{M_{i}}$ (the number of predictions included), and $r_{k}\;\left(x_{i},\Omega^{M_{j}}\right)$ corresponds to the Euclidean distance to the *k*^*t* *h*^ nearest neighbor of *x*_*i*_ in $\Omega_{M_{j}}$. Note that the backslash indicates excluding an element from the set. Using this equation, the JSD can straightforwardly be computed for all possible combinations of experiments and used to rank according to how well these experiments would discriminate between the models. A larger value for the JSD indicates an informative experiment. The last step involves sampling several combinations of measurements and determining the set of experiments which have the greatest JSD. The proposed methodology is depicted in Figure 4.

### Testing the method: numerical experiments

To demonstrate the method, a series of simulation studies are performed. Since we know which model generated the data, it is possible to compare to the Bayes factor pointing to the true model. After generating an initial data set using the true model, PPDs are sampled for each of the competing models. As the design variable, we consider the timing of a new measurement. Hence, we look at differences between the predictive distributions belonging to the different models at different timepoints. We use a sample of simulated observables at specific timepoints to compute JSD estimates between the different models. We thereby test whether the JSD estimate can be used to compare different potential experiments. The new experimental data is subsequently included and the JSD compared to the change in Bayes factor in favor of the *correct model*. Note that this new Bayes factor depends on the experimental outcome and that this approach results in a distribution of predicted Bayes factors. A large change in Bayes factor indicates a useful experiment.

#### Analytic models

The method is applied to a number of linear regression models. Linear regression models are models of the form:

(12) $$y(t)=\overset{L}{\underset{i=1}{\sum }}\theta_{i}B_{i}(t)+\varepsilon$$

where $\vec{\theta }$ represents a parameter vector and **B** constitutes a design matrix with basis functions *B*_*i*_(*t*). Given that *σ*, the standard deviation of the Gaussian observation error *ε*, is known and the prior distribution over the parameters is a Gaussian with standard deviation *ξ*, the mean and covariance matrix of the posterior distribution can be computed analytically (see Additional file 1: section S4). Using linear models avoids the difficult numerical integration commonly required to compute the Bayes factor and makes it possible to perform overarching Monte Carlo studies on how these Bayes factors adjust upon including new experimental data. The analytical expressions make it possible to compare the JSD to distributions of the actual Bayes factors for model selection.

#### Non-linear biochemical networks

**Figure 5:**
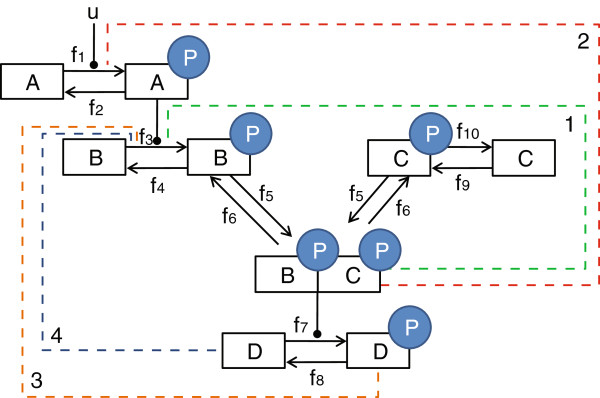
**Non-linear biochemical network hypotheses.** Models used to test the method. Here *p* refers to a phosphorylated species. The dashed lines indicate the different hypotheses regarding the negative feedback mechanisms in each of the models. Here feedback 1 corresponds to the true data generating model. Data of *Bp* and *Dp* was used for inference.

To further test the methodology, a series of artificial models based on motifs often observed in signaling systems [58,59] were implemented (see Additional file 1: section S5 for model equations). Artificial data was simulated for *M*_1_. Subsequently, inference was performed for all four competing topologies. The difference between each of the models was the origin and point of action of the feedback mechanism (see Figure 5).

Each of the artificial models was able to describe the measured data to an acceptable degree. We used a Gamma distribution with *α*=1 and *β*=3 for the prior distributions of the parameters. This prior is relatively non-informative (allowing a large range of parameter values), while not being so vague that the simplest model is always preferred (Lindley’s paradox [40]). Data was obtained using *M*_1_. Observables were *Bp*, of which three replicates were measured, and *Dp*, of which two replicates were measured. These were measured at *t*=[ 0,2,5,10,20,40,60,100]. All replicates were simulated by adding Gaussian white noise with a standard deviation of 0.03. The parameter values corresponding to the true system were obtained by running Monte Carlo simulations until a visible overshoot above the noise level was observed. Parameter inference was performed using population MCMC with the noise *σ* as an inferred parameter. As design variables we consider both the choice of which observable(s) to measure and the time point(s) of the measurement.

### Computational implementation

All algorithms were implemented in Matlab (Natick, MA). Numerical integration of the differential equations was performed with compiled MEX files using numerical integrators from the SUNDIALS CVode package (Lawrence Livermore National Laboratory, Livermore, CA). Absolute and relative tolerances were set to 10^−8^ and 10^−9^ respectively. MCMC was performed using a population MCMC approach using *N*_*T*_=40 chains with a temperature schedule given by $T_{n}={\left(\frac{N_{T}}{n}\right)}^{4}$[52]. This also permitted computation of the Bayes factors between the non-linear models by means of thermodynamic integration. The Gaussian proposal distribution for the MCMC was based on an approximation to the Hessian computed using a Jacobian obtained by simulating the sensitivity equations. After convergence, the chain was thinned to 10000 samples. Since the number of experiments designed simultaneously (and therefore the dimensionality of the prediction vectors) was reasonably small (*N*_*samples*_>>2^*k*^), the kNN search was performed using k-d trees [60]. The figures in this paper were determined using *k*=10.

## Results and discussion

### Analytic models

A series of experiments were performed using linear regression models. To demonstrate the method, consider the following four competing models, where *M*_3_ is used to generate the data:

(13) $$\begin{array}{ll} y_{M_{1}} & =\theta_{1}t\; \end{array}$$

(14) $$\begin{array}{ll} y_{M_{2}} & =\theta_{1}t+\theta_{2}t^{2}\; \end{array}$$

(15) $$\begin{array}{ll} y_{M_{3}} & =\theta_{1}t+\theta_{2}t^{2}+\theta_{3}\text{sin}\;\left(\frac{1}{5}t^{3}\right) \end{array}$$

(16) $$\begin{array}{ll} y_{M_{4}} & =\theta_{1}t+\theta_{2}t^{2}+\theta_{3}\text{sin}\;\left(\frac{1}{5}t^{3}\right)+\theta_{4}\text{sin}\;\left(2t\right)t\; \end{array}$$

The presence of sine waves in *M*_3_ and *M*_4_ elicits particularly noticeable patterns in the optimal experiment design matrices. *D* equidistantly sampled time points were generated as data (including Gaussian additive noise *σ*). To make sure that the model selection was unsuccessful *a priori*, these were sampled in a region where the models roughly predict the same behavior. Initially, the Bayes factors were log10(*B*_31_)=2.0439 (decisive), log10(*B*_32_)=−0.0554 (pointing to the wrong model) and log10(*B*_34_)=0.4658 (‘not worth more than a bare mention’ [51]). PPDs were generated for each of the models and used to compute credible predictive intervals that enclose 95% of the predictive density. Bayes factors were computed for each of the models. Since the aim of the design is to successfully select between the models after performing new experiments, the change in Bayes factor in favor of the *true* underlying model was computed. Since the experimental outcome is not known *a priori*, a distribution of Bayes factors is predicted:

(17) $$\begin{array}{c} \;\Delta (B_{\text{ab}}):=E\;\left[\log_{10}\;\left(\frac{p(y^{D},y_{n}^{D}|\;M_{a})}{p(y^{D},y_{n}^{D}|\;M_{b})}\right)\right]-\log_{10}\;\left(\frac{p(y^{D}|\;M_{a})}{p(y^{D}|\;M_{b})}\right). \end{array}$$

**Figure 6:**
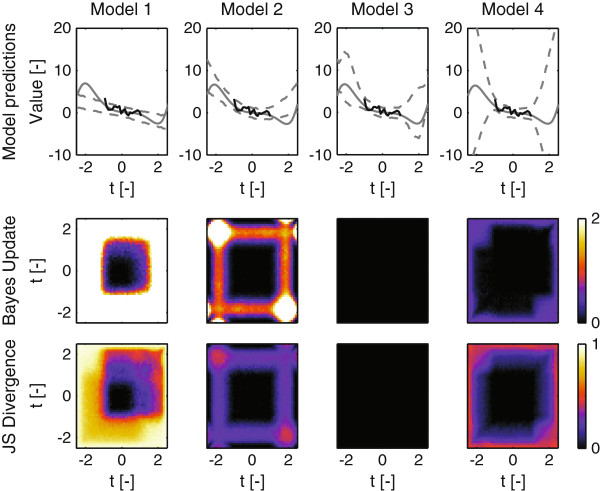
**Comparing the Jensen Shannon divergence to the Bayes factor updates for regression models.** Top row: Solid gray line indicate the ’true’ system response to the true parameters which is the same in each graph. The solid black line indicates the data generated by model three, which was used to determine the posterior predictive distribution on the four models. Dashed lines indicate the inferred credible intervals of the Posterior Predictive Distribution for the model corresponding to that column. Second row: Mean of the predicted change in Bayes factor in favor of model three after incorporating two additional data points from the posterior predictive distribution of the true model (average of 100 repetitions). Coordinate along each axis reflects the point *t* at which the measurement is performed. Bottom row: Jensen Shannon Divergence between the Posterior Predictive Distributions of *M*_3_ and the model that column number corresponds to. Here *M*_3_ corresponds to the model which generated the data. Note that the graphs for model three are black by definition.

The expectation is taken with respect to new realizations of the data $y_{n}^{D}$. Note that such an overarching estimation would be computationally intractable for a non-linear model. New experiments can be simulated in two ways. Either by using the correct model with the true parameter values and adding measurement noise or by taking samples from the posterior predictive distribution of the correct model (*ΔBabB*). In practice these ‘true’ parameter values are not known and the predictive distribution of the measurement based on the posterior samples provides the best obtainable estimate given the current parameter uncertainty. The change in Bayes factor (in favor of the correct model) was compared to the Jensen Shannon divergence between the competing models. Large predicted changes indicate that the experiment would result in a successful selection. As for the JSD, a large value indicates a large distance between the joint predictive distributions, marking the measurement as useful. See Figure 6 for an example of the analysis results. As shown in the different panels, different models parameterized on the same data, result in different posterior predictive distributions (dotted lines in the top row). When comparing model 1 and 3 (the true model), we can see that differences in predictions occur mostly beyond the time range previously measured. Whereas model 1 predicts a straight line, the true underlying system deviates from a single line. Consider two new measurements. From the differences in PPDs, it is clear that measuring beyond the region where data is available would lead to a decisive choice against model 1 as corroborated by the large Bayes factor updates shown in the left plot on the middle row (*B*_13_). It can also be seen that the PPDs differ more for negative time than positive time. Therefore the area which is decisive is larger for negative time. The JSDs follow this same pattern. The PPD for model 2 is less different from the PPD the true model would have generated. For the simulations coming from model 2, we can see that the value for positive and negative time is correlated. For model 3, these prediction values are negatively correlated. Consequently, performing one measurement for negative time and one for positive time would lead to the largest discriminatory power.

**Figure 7:**
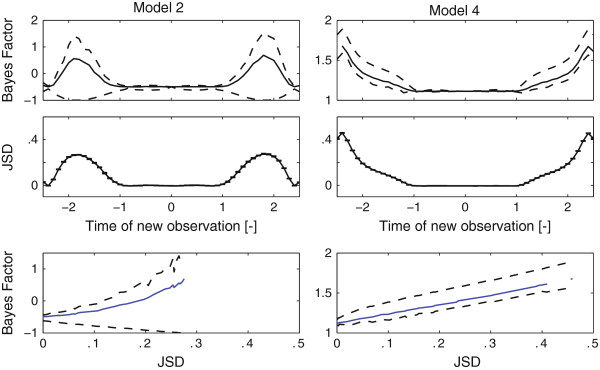
**Comparing the Jensen Shannon Divergence to the updated Bayes factor.** Inference was performed based on data generated from the true model. Bayes factor updates pointing to the correct model were calculated given a new measurement which was based on the inferred posterior predictive distribution of the true model. Top row: Predictive distribution of updated Bayes factor in favor of the correct model with associated 67% credible intervals (dashed) for two models. Second row: Jensen Shannon divergence between the relevant Posterior Predictive Distributions. Bottom row: Relation between the updated Bayes factors and the JSD. Note that the relation is nearly monotonic indicating its usefulness as a predictor of experimental efficacy.

The JSD agrees well with the actual Bayes factor updates as shown in the third row of Figure 6. Interestingly, all the designs based on the JSD result in designs that would effectively discriminate between the true model and its competitors without having to specify a true model *a priori*. Subsequently, a Monte Carlo study was performed where a large number of random models were generated and compared. Plotting the relationship between the updated Bayes factors upon a new experiment and the corresponding JSD typically reveals a monotonic relationship that underlines its usefulness as a design criterion (see Figure 7 for two typical examples). These analyses were performed for a large number of randomly generated linear models. The Spearman correlation coefficient between the JSD and the expected Bayes factor averaged at 0.91 for the experiments we performed (see Additional file 1: section S6).

### Nonlinear models

**Figure 8:**
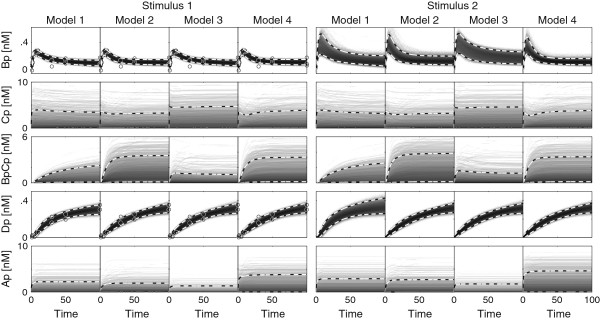
**Posterior predictive distributions of the non-linear ODE models.** The first five predictions in the left panels correspond to the same experimental condition as during the original inference (stimulus 1) while the second five predictions correspond to a different stimulus (stimulus 2). Note that the differences between the different distributions are barely visible. Data is denoted in circles.

**Figure 9:**
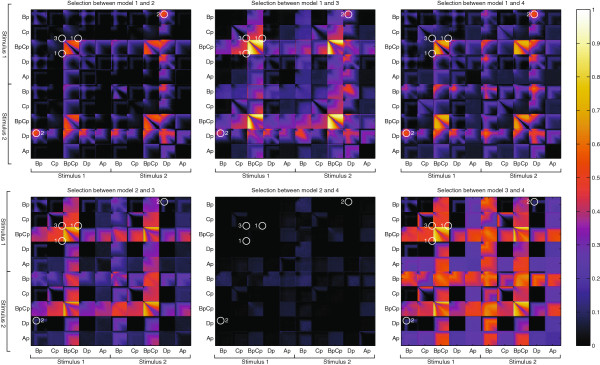
**Jensen Shannon divergences for each model comparison in the nonlinear case.** Each axis represents a single measurement. Each tile corresponds to a combination of two state variables where the space within each tile corresponds to the actual time point at which the state variable is measured. The first five predictions correspond to the same experimental condition as during the original inference (stimulus 1) while the second five predictions correspond to a different stimulus (stimulus 2). The circles denote the different experiments that were performed.

In Figure 8, we show the different predictions after performing model inference. Two sets of PPDs were simulated for two experimental conditions. These sets mimic two different concentrations of a signaling molecule, and have been implemented by setting the stimulus *u* to either 1 or 2. To test the effect of measuring multiple predictions, divergence estimates were computed for a large number of different combinations of two measurements. These results are shown in Figure 9. Each subplot corresponds to a different model comparison. The axis of each subplot is divided into ten sections corresponding to different predictions. Within each section, the axis represents time. The color value indicates the JSD, where a large value indicates a lot of separation and therefore a good measurement. Note the bright squares corresponding to the concentration of *BpCp* in each of the models. These high efficacies are not surprising considering that the PPDs show large differences between the models for these concentrations (See Figure 8). Also noticeable is that many of the experiments on the same predictions reveal dark diagonals within each tile. Measuring the same concentration twice typically adds fewer predictive constraints than measuring at two different time points, unless the second measurement is performed using a different concentration of signaling molecule (note how the diagonal lights up on the combination of measuring *BpCp* in condition 1 and 2 when selecting between model 3 and 4). Interestingly, measuring certain states during the overshoot is highly effective (*Bp* and *Dp* for any comparison), while the overshoot is less informative for others (*Bp* under stimulus 1 and 2 for discriminating between *M*_1_ and *M*_2_). All in all, the information contained in such a matrix is very valuable when it comes to selecting from a small list of experiments. For example, we can also see that considering the current predictive distributions, model 2 and 4 can barely be distinguished. This implies that in order to actually distinguish between these two, a different experiment is required. Such a new experiment could, again, be evaluated by generating a new competing set of PPDs.

To test the results, *in silico* experiments have been performed by simulating new data from the true model and determining the Bayes factor change upon including this data. Bayes factors were estimated using thermodynamic integration (see Additional file 1: section S2). The calculation of each set of four marginal likelihoods took about 6 days of wall-clock time on an Intel i7 CPU (2.93 GHz) with MATLAB R2010a. To validate the method, experiments are selected where differences between models are expected. The following experiments were performed: 

● 1. Steady state *Cp* and *BpCp* concentration

● 2. *Bp* and *Dp* during the peak in the second condition (*u*=2)

● 3. Steady state *Cp*

Experiment 1 should differentiate between *M*_1_ and *M*_3_ (*D*_13_≈0.49), but not between *M*_1_ and *M*_2_ or *M*_1_ and *M*_4_. Experiment 2 should give discriminatory power for all models (*D*_12_≈0.48,*D*_13_≈0.54,*D*_14_≈0.61). Experiment 3 should not provide any additional discriminatory power at all. The results of these analyses are shown in Table 1. As predicted, experiment 1 leads only to an increase in discriminatory power between model *M*_1_ and *M*_3_. Experiment 2 improves the discriminatory power between all the models, while experiment 3 even reveals a decrease in discriminatory power for model 1 and 2. Noteworthy is also the large variance observed for experiment 3, which is likely related to the large variance in the steady state predictions of *Cp*. Again, the predictions based on the JSD are well in line with the Bayes factors obtained.

**Table 1:** Sample table title

***D***_**12**_	***Δ*** ***B***_**12**_	***D***_**13**_	***Δ*** ***B***_**13**_	***D***_**14**_	***Δ*** ***B***_**14**_
0.03	0.06±0.19	0.49	0.32±0.39	0.05	−0.07±0.36
0.48	0.26±0.14	0.54	0.72±0.36	0.61	0.43±0.38
−0.06	−0.49±0.73	−0.01	−0.35±0.68	−0.04	0.32±0.54

JSD and change in Bayes factors denoted as mean ± standard deviation for each of the reported experiments (n = 3).

## Conclusions

This paper describes a method applicable to performing experiment design with the aim of differentiating between various hypotheses. We show by means of a simulation study on analytically tractable models that the JSD is approximately monotonically related to the expected change in Bayes factor in favor of the model that generated the data (considering the current uncertainty in its parameters). This monotonic relation is useful, because it implies that the JSD can be used as a predictor of the change in Bayes factor. The applicability to non-linear models of biochemical reaction networks was demonstrated by applying it to models based on motifs previously observed in signaling networks [58,59]. Experiments were designed for distinguishing between different feedback mechanisms.

Though forecasting a predictive distribution of Bayes factors has been suggested [61], the implicit penalization of model complexity could have adverse consequences. The experiment design could suggest a measurement where the probability densities of two models overlap. When this happens, both models can describe the data equally well, which leads to an implicit penalization of the more complex model (since it allows for more varied predictions due to its added freedom). This penalization can then be followed by subsequent selection (of the simpler model). Though a decisive selection occurs, such an experiment would not provide additional insight however. In [61], this is mitigated by determining the evidence in favor of a more complex model. Moreover, computing the predictive distributions of Bayes factors required for this approach is computationally intractable for non-linear models that are not nested. By focusing on differences in predictive distributions, both these problems are mitigated, making it is possible to pinpoint where the different models predict different behavior. Aside from their usefulness in model selection, such predictive differences could also be attributed to the different mechanisms present in the different models. This allows for follow-up studies to investigate whether these are either artificial or true system behavior.

A complicating factor in this method is the computational burden. The largest challenge to overcome is to obtain a sample from the posterior parameter distribution. Running MCMC on high dimensional problems can be difficult. Fortunately, recent advances in both MCMC [19,62] as well as approximate sampling techniques [39,48,63,64] allow sampling parameter distributions of increasingly complex models [14,34-38]. The bottleneck in computing the JSD resides in searching for the *k*^*th*^ nearest neighbor. A subproblem which occurs in many different problems and for which computationally faster solutions exist [65,66]. An attractive aspect of this methodology is that it is possible to design multiple experiments at once. However, the density estimates typically become less accurate as the number of designed experiments increases (see Additional file 1: section S8). Therefore, we recommend starting with a low number of experiments (two or three) and gradually adding experiments while the JSD is low. Density estimation can also be problematic when the predictions vary greatly in their dispersion. When considering non-negative quantities such as concentrations, log-transforming the predictions may alleviate problems. Finally, the number of potential combinations of experiments increases exponentially with the number of experiments designed. It is clear that this rapidly becomes infeasible for large numbers of experiments. However, it is not necessary to fill the entire experimental matrix and techniques such as Sequential Monte Carlo sampling could be considered as an alternative to more effectively probe this space. We revert the reader to Additional file 1: section S7 for a proof of principle implementation of such a sampler.

One additional point of debate is the weighting of each of the models in the mixture distribution used to compute the JSD. It could be argued that it would be more sensible to weight models according to their model probabilities by determining the integrated likelihoods of the data that is already available. The reason for not doing this is two-fold. Firstly, the computational burden this adds to the experimental design procedure is significant. More importantly however, the implicit weighting in favor of parsimony could strongly affect the design by removing models which are considered unnecessarily complex at this stage of the analysis. When designing new experiments, the aim is to obtain measurements that allow for optimal discrimination between the predictive distributions under the different hypotheses. Optimal discrimination makes it sensible to consider the models equally probable *a priori*.

The method has several advantages that are particularly useful for modeling biochemical networks. Because the method is based on sampling from the posterior parameter probability distribution, it is particularly suitable when insufficient data is available to consider Gaussian parameter probability distributions or model linearisations. Additionally, it allows incorporation of prior knowledge in the form of prior parameter probability distributions. This is useful when the available data contains insufficient constraints to result in a well defined posterior parameter distribution. Because the design criterion is based on predictive distributions and such distributions can be computed for a wide range of model quantities, the approach is very flexible. In biochemical research, *in vivo* measurements are often difficult to perform and practical limitations of the various measurement technologies play an important role. In many cases measurements on separate components cannot be performed and measurements result in indirect derived quantities. Fortunately, in the current framework such measurements can be used directly since distributions of such experiments can be predicted.

Moreover, the impact of specific combinations of experiments can be assessed by including them in the design simultaneously which reveals specific combination of measurements that are particularly useful. This way, informative experiments can be distinguished from non-informative ones and the experimental efforts can be targeted to discriminate between competing hypotheses.

## Availability

Source code is available at: http://cbio.bmt.tue.nl/sysbio/software/pua.html.

## Competing interests

The authors declare that they have no competing interests.

## Authors’ contributions

JV developed the method, performed the analyses, and wrote the paper. CT, PH and NR contributed to the computational analysis, interpretation of the results and revised the paper. All authors read and approved the final manuscript.

## Supplementary Material

Additional file 1 **Supplementary Information.S1.** Additional information regarding the MCMC Sampler. **S2.** Thermodynamic integration. **S3.** Mutual information. **S4.** Analytical expressions for the linear models. **S5.** Nonlinear model equations. **S6.** Spearman correlation JSD and Bayes factor. **S7.** Sampling bigger design matrices. **S8.** Choosing the size parameter for the density estimation. Click here for file
