# Supplementary material for: Optimal experiment design for model selection in biochemical networks
Source: BMC Syst Biol. 2014 Feb 20;8:20. doi: 10.1186/1752-0509-8-20 (PMC3946009; doi:10.1186/1752-0509-8-20)
Supplement: Additional file 1 — Supplementary Information.S1. Additional information regarding the MCMC Sampler. S2. Thermodynamic integration. S3. Mutual information. S4. Analytical expressions for the linear models. S5. Nonlinear model equations. S6. Spearman correlation JSD and Bayes factor. S7. Sampling bigger design matrices. S8. Choosing the size parameter for the density estimation. [file 1752-0509-8-20-S1.pdf]

Optimal Experiment Design for Model Selection in Biochemical Networks (Supplement)

J Vanlier, CA Tiemann, PAJ Hilbers, NAW van Riel

October 22, 2013

The supplementary materials consist of the following sections:

- S1. Additional information regarding the MCMC Sampler
- S2. Thermodynamic integration
- S3. Mutual information
- S4. Analytical expressions for the linear models
- S5. Nonlinear model equations.
- S6. Spearman correlation JSD and Bayes factor
- S7. Sampling bigger design matrices.
- S8. Choosing the size parameter for the density estimation

S1. MCMC sampler

This section contains some of the implementational details of the Markov Chain Monte Carlo (MCMC) sampler used in this paper. MCMC is a technique used to obtain samples from probability distributions known only up to a normalizing factor. Given that $p(\vec{\theta})$ is a non-negative integrable function, the Metropolis Hastings algorithm will provide a sequence of samples (also known as chain) whose equilibrium distribution is proportional to $p(\vec{\theta})$ using only evaluations of $p(\vec{\theta})$. If the chain is irreducible and aperiodic then the chain will settle down to a limiting (ergodic) distribution. Irreducibility requires that it must be possible to get from any two possible states of the system to the other in a finite number of steps. A sufficient condition to ensure that $p(\vec{\theta})$ is the limiting distribution is that the sampler satisfies detailed balance (1) where $\pi(.)$ corresponds to the invariant distribution and $p(x, y)$ corresponds to the distribution used for proposing the next step (the proposal distribution). This property ensures that the chain is reversible (two sides are equal).

$$\pi(x)p(x, y) = \pi(y)p(y, x) \tag{1}$$

The Metropolis algorithm ensured chain ergodicity by using only symmetric proposals (where the probability of a forward and backward move should be equal, i.e. the proposal distribution does not change). A generalization by Hastings lead to the an additional term in the acceptance probability which ensures detailed balance for non-symmetric proposal mechanisms. The resulting algorithm, named the Metropolis-Hastings algorithm is generally considered as the workhorse of MCMC methods. The algorithm proceeds by iteratively performing a number of steps:

- 1. Generate a sample $\vec{\theta}_{new}$ by sampling from a proposal distribution based on the current state
- 2. Compute the likelihood of the data $L(\mathbf{y^D}|\vec{\theta}_{new})$ and calculate $p(\vec{\theta}_{new}|\mathbf{y^D}) = L(\mathbf{y^D}|\vec{\theta}_{new})p(\vec{\theta}_{new})$, where $p(\vec{\theta}_{new})$ refers to the prior density function.
- 3. Draw a random number γ from a uniform distribution between 0 and 1 and accept the new step if $\gamma < min\left(\frac{p(\vec{\theta}_{n+1}|\mathbf{y^D})q(\vec{\theta}_{n+1}\rightarrow\vec{\theta}_n)}{p(\vec{\theta}_n|\mathbf{y^D})q(\vec{\theta}_n\rightarrow\vec{\theta}_{n+1})}, 1\right)$.

The ratio of q is known as the Hastings correction and ensures detailed balance, a sufficient condition for the Markov Chain to converge to the equilibrium distribution. It corrects for sampling biases resulting from non-symmetric proposal mechanisms. It corrects for the fact that the probability density going from parameter set $\vec{\theta}_n$ to $\vec{\theta}_{n+1}$ and $\vec{\theta}_{n+1}$ to $\vec{\theta}_n$ is unequal when the proposal distribution depends on the current parameter set. It is defined as the ratio between the proposal densities associated with going from n to $n+1$ and $n+1$ to n. The apparent simplicity of the algorithm makes it conceptually attractive. Naive approaches can lead to MCMC samplers that converge slowly and/or stay in the local neighborhood of a local mode [1].

Proposals

Regarding the proposal distribution, we employ an adaptive Gaussian proposal distribution whose covariance matrix is based on a quadratic approximation to the probability density [3]. This matrix is computed by taking the inverse of an approximation to the Hessian matrix of the model under consideration. Such adaptation to the local geometry of the problem results in taking larger steps in directions where the probability density does not change much (improving efficiency of the sampler). Such a Gaussian distribution is characterized by a positive definite covariance matrix $\mathbf{\Sigma}$, the number of dimensions d and the vector of mean values $\vec{\mu}$ (2). Sampling from such a distribution is straightforward. Compute a decomposition such that $\mathbf{R}\mathbf{R}^T = \mathbf{\Sigma}$. Subsequently draw a vector $\vec{z}$ of N independent normal variates.

$$\frac{1}{(2\pi)^{\frac{d}{2}}\sqrt{|\mathbf{\Sigma}|}} e^{-\frac{1}{2}(\vec{x}-\vec{\mu})^T \mathbf{\Sigma}^{-1}(\vec{x}-\vec{\mu})} \tag{2}$$

This approach allows the sampler to follow the curvature of the distribution naturally.

In the current implementation the normal distribution is centered around the current point therefore the expression for the next iteration becomes $\vec{x}_{new} = \vec{x} + \mathbf{R}\vec{z}$. If the proposal distribution depends on the current state (asymmetric proposals), then the proposal needs to be corrected for the imbalance in proposal densities in the two directions using the Hastings term, which can be calculated for a multivariate Gaussian proposal distribution as (3).

$$Q(\vec{\theta}_n \to \vec{\theta}_{n+1}) = \frac{1}{\sqrt{|\mathbf{\Sigma_a}|}} e^{-\frac{1}{2}(\vec{\theta}_{n+1}-\vec{\theta}_n)\mathbf{\Sigma_a}^{-1}(\vec{\theta}_{n+1}-\vec{\theta}_n)^T} \tag{3}$$

Calculating the true Hessian is costly and numerically challenging, which is why an approximation based on the model sensitivities is used. Depending on the model, these can either be computed by solving the sensitivity equations, or by means of finite differences (for which *strict tolerances* are required to ensure reliable derivatives). Additionally, we include including non-uniform priors (when available) in the Hessian approximation, by including their respective derivatives in the approximation of the sensitivity matrix $\mathbf{S}$. Estimating the standard deviations of the noise on the data adds the requirement of having to include part of the normalizing constant of the likelihood K. This is incorporated as an additional element in the vector of squared residuals. The 'sensitivity' of this residual is subsequently given by $\sqrt{.5||dK/d\vec{\theta}||}$ which worked well for all the investigated problems. This Hessian approximation is subsequently decomposed using the singular value decomposition (4), where $\mathbf{S}$ is a diagonal matrix containing the singular values. The decomposition delivers a matrix of singular vectors U and a diagonal matrix with associated singular values. Large singular values of the Hessian matrix correspond to well constrained directions, while low values correspond to poorly constrained directions in parameter space.

$$\mathbf{H} = \mathbf{U}\mathbf{S}\mathbf{V}^T \tag{4}$$

In practical cases, some directions in parameter space can be so poorly constrained that this leads to a (near) singular Hessian (some singular values near zero). As a result, the proposal distribution will become extremely elongated in these directions, leading to proposals where parameters take on extreme values and acceptance ratios decline due to integration failures or rejections due to low probability density. One approach to avoid such numerical difficulties is to set singular values below a certain threshold to a specific minimal threshold (prior to inversion).

The avoid the computation of costly inverses, we compute the sampling matrix directly from the SVD using (5). Here s corresponds to a problem specific (tuned) scaling factor, T to the temperature N_{dim} to the number of parameters.

$$\mathbf{R} = \frac{s\sqrt{T}}{\sqrt{N_{dim}}}\mathbf{V}\sqrt{\mathbf{S}^{-1}} \tag{5}$$

The inverse required for the Hastings correction can subsequently be computed as (6). Since the determinant only appears in ratios, the linear scaling needs not be explicitly calculated since it will cancel out due to the dimensionality of the problem remaining constant (7). Therefore the ratio is computed as a product of the ratios of the singular values.

$$\mathbf{\Sigma}^{-1} = \frac{N_{dim}}{s^2 T}\mathbf{V}\mathbf{S}\mathbf{V}^{\mathbf{T}} \tag{6}$$

$$det(\mathbf{\Sigma}) = \left(\frac{N_{dim}}{s^2 T}\right)^{-N_{dim}} \prod \frac{1}{S_{ii}} \tag{7}$$

Parameter representation

In order to deal with the large difference in scales, certain parameters can be considered in log-space. Note however, that the prior distribution is generally not invariant of the way the model is parameterized. The transformation between parameters can be described by the matrix of partial derivatives with respect to the equations which transform the parameters from one parameterization to another (the Jacobian of the transformation). In order to calculate a prior distribution that is equivalent in terms of inference under a different parameterization, one needs to compute the absolute value of the determinant of the Jacobian of the transformation. This corrects for the stretching and compression of the distribution due to the reparameterization (8).

$$P(f(\vec{\theta})) = P(\vec{\theta}) \left| \begin{bmatrix} \frac{df(\theta_1)}{d\theta_1} & \cdots & \frac{df(\theta_1)}{d\theta_n} \\ \vdots & \ddots & \vdots \\ \frac{df(\theta_n)}{d\theta_1} & \cdots & \frac{df(\theta_n)}{d\theta_n} \end{bmatrix} \right| \tag{8}$$

In the case where we perform the MCMC in logarithmic space, we obtain the following expression (9) to be included in the acceptance probability.

$$\frac{|J(\theta^a)|}{|J(\theta^b)|} = \prod_i^{N_{pars}} \left(\frac{\theta_i^b}{\theta_i^a}\right) \tag{9}$$

For the Hessian based approach, the proposals can subsequently be generated using the equation in (10). Where the Hessian approximation in log space is computed by applying the chain rule (11).

$$\vec{\theta}_{n+1} = \vec{\theta}_n e^{N(0,\mathbf{\Sigma}_{\log})} \tag{10}$$

$$\frac{\delta^2 L}{\delta \log \theta_i \delta \log \theta_j} = \frac{\delta^2 L}{\delta\theta_i \delta\theta_j}\theta_i\theta_j \tag{11}$$

Metropolis coupled MCMC

To improve mixing for multi-modal distributions Metropolis Coupled MCMC was implemented [1]. Here multiple chains are run simultaneously with interactions after each iteration. Each chain can optionally run at a different temperature T (12).

$$P_T(\mathbf{y^D}|\vec{\theta}, T) = P(\mathbf{y^D}|\vec{\theta})^{\frac{1}{T}} \tag{12}$$

Samples are exchanged between the chains using switch moves. These are either performed by randomly selecting two adjacent temperatures and computing an Metropolis-Hastings step using the acceptance ratio given in (13). Or by assigning one 'mother-chain' which is involved in every interaction.

$$\alpha < min\left(\frac{P(\mathbf{y^D}|\vec{\theta}_{new})^{\frac{1}{T_n}} P(\mathbf{y^D}|\vec{\theta}_n)^{\frac{1}{T_{new}}}}{P(\mathbf{y^D}|\vec{\theta}_n)^{\frac{1}{T_n}} P(\mathbf{y^D}|\vec{\theta}_{new})^{\frac{1}{T_{n+1}}}}\right) \tag{13}$$

The multiple chains define a joint probability space. Implementationally, multiple instances of the parameter vector and probability density values are concatenated. Each copy corresponding to a different chain. Regular MCMC updates are performed per parameter group, while switch moves enable the sampler to switch the parameters between two groups. One advantage of this approach is that the cost function will flatten out for higher temperatures. Therefore chains at higher temperatures are able to traverse the solution space more freely.

Autocorrelation

An MCMC sampler will generate samples that are often highly correlated. The autocorrelation function of an observable or parameter can be evaluated. Such correlations decrease approximately exponentially suggesting that the chain effectively loses its correlation as it progresses. Therefore, the sample taken a

certain number of iterations later is no longer closely related to the initial one. Slowly decaying autocorrelations are a warning sign that the chain is mixing slowly for that observable or parameter. Because of the correlated nature of MCMC chains, results are often thinned. Here one employs subsampling by only using every m^{th} iteration. Such subsampling can be considered when computing predictions using samples obtained via MCMC is costly compared to running the chain [2] and/or the autocorrelation function decreases slowly.

Estimates of the ESS were obtained using the initial monotone sequence estimator [2]. The ESS is computed from the empirical autocorrelation $\rho(t)$. One first defines the sum of adjacent pairs of autocorrelations $\phi(t) = \rho(2t) + \rho(2t+1)$. Subsequently one enforces monotonicity by setting each subsequent $\phi(t)$ to the minimum of the preceding ones. Subsequently d is chosen such that $\phi(t)$ is positive for $1...d$. The ESS is then given by (14) where M denotes the number of samples in the MCMC chain.

$$ESS = \frac{M}{-1 + 2\sum_{t=0}^{d} \phi(t)} \tag{14}$$

Computational methods

All of the algorithms were implemented in Matlab (Natick, MA). Numerical integration was performed using compiled MEX files using numerical integrators from the SUNDIALS CVode package (Lawrence Livermore National Laboratory, Livermore, CA). Absolute and relative tolerances were set to 10^{-8} and 10^{-9} respectively. Integration time for a single integration was allowed to be 10 seconds at most after which an integration is assumed to fail and a large error is returned. Throughout the analysis integration failures were carefully monitored.

In order to attain an adequate acceptance rate and good mixing, the proposal scaling was determined during an initial tuning stage. This tuning was performed by running many short chains (100 iterations each), targeting an acceptance rate between 0.2 and 0.4. If the acceptance rate was high, 10% was added to the scale, while 10% was subtracted in the case where the acceptance was too low. Interestingly the chains at higher temperatures had very similar acceptance rates once started.

S2. Thermodynamic integration

In order to estimate the Bayes factor, the marginal likelihood needs to be computed. For a single model this quantity looks as follows

$$p(\mathbf{y}^{\mathbf{D}}|M) = \int p(\mathbf{y}^{\mathbf{D}}|M,\vec{\theta})p(\vec{\theta}|M)d\vec{\theta} \tag{15}$$

One approach to determine this marginal likelihood is to draw random samples $\vec{\theta}$ from the prior and average the likelihood values for those samples. However, due to the fact that most samples will be taken in regions of low likelihood, this method is highly inefficient. Another approach would be to employ importance sampling. One tempting approach here is to use the posterior density function as an importance sampling function leading to the following Monte Carlo Estimate

$$\left(\frac{1}{N}\sum_{i=1}^{N} p(\mathbf{y}^{\mathbf{D}}|M,\vec{\theta})^{-1}\right)^{-1} \tag{16}$$

but this estimate suffers from both numerical instability as well as severe bias [1]. The alternative and more stable approach is to use *thermodynamic integration*, where the problem is recast into a problem involving integration over various intermediate distributions which are defined as follows

$$p_T(\vec{\theta}|\mathbf{y}^{\mathbf{D}}) = \frac{p(\mathbf{y}^{\mathbf{D}}|M,\vec{\theta})^T p(\vec{\theta})}{z(T)} \tag{17}$$

with

$$z(T) = \int p(\mathbf{y}^{\mathbf{D}}|M,\vec{\theta})^T p(\vec{\theta}|M)d\vec{\theta} \tag{18}$$

Consider the following

$$\int_0^1 \frac{d}{dT}\log(z(T))dT = \int_0^1 \frac{1}{z(T)}\frac{d}{dT}z(T)dT = \log(Z(1)) - \log(Z(0)) \tag{19}$$

It can be seen that $Z(1)$ corresponds to the marginal likelihood, while $Z(0)$ corresponds to the integrated prior (which is equal to 1). Consider that

$$z(T) = \int e^{\log(p(\mathbf{y}^{\mathbf{D}}|M,\vec{\theta}))T} p(\vec{\theta}|M)d\vec{\theta} \tag{20}$$

from which it follows that

$$\log(Z(1)) = \int_0^1 \int \frac{p(\mathbf{y}^{\mathbf{D}}|M,\vec{\theta})^T p(\vec{\theta}|M)}{z(T)} \log(p(\mathbf{y}^{\mathbf{D}}|M,\vec{\theta}))d\vec{\theta}dT \tag{21}$$

Therefore, after obtaining samples at different intermediate distributions spaced between $T = 0$ and $T = 1$, this integration can be performed numerically by summing the expected value of the log likelihoods at different temperatures.

$$\hat{p}(\mathbf{y}^{\mathbf{D}}|M) = \sum_{q=1}^{T_{max}} \frac{1}{N_{samples}} \sum_{i=1}^{N_{samples}} \log\left(p(\mathbf{y}^{\mathbf{D}}|M, \vec{\theta}_i^{T_q})\right) \tag{22}$$

Where T_q and $\vec{\theta}_i^{T_q}$ are the temperature and samples associated with chain q. $N_{samples}$ indicates the number of samples per chain. The temperature schedule used in this study is given by

$$T_q = \left(\frac{N_T}{q}\right)^{\gamma} \tag{23}$$

with $N_T = 40$ and $\gamma = 4$. For further information see [1].

S3. Mutual information

Mutual information is a quantity that measures the mutual dependence between two random variables

$$I(X,Y) = \int_Y \int_X p(x,y) \log \left(\frac{p(x,y)}{p(x)p(y)} \right) dxdy \tag{24}$$

It measures the amount of information shared between two random variables. A large mutual information implies that knowing the value of one of the variables reduces the uncertainty in the other. If two random variables are independent, their mutual information is zero. If entropy

$$H(X) = \int_X p(x) \log(p(x))dx \tag{25}$$

is considered as a measure of uncertainty of a random variable, the mutual information can be expressed as follows

$$I(X,Y) = H(X) - H(X|Y) \tag{26}$$

Informally, the mutual information reflects how much the uncertainty in one random variable is reduced by knowing about another. Assuming that the models are equally probable a-priori, averaging the predictive distribution of a new measurement over the different models corresponds to the additive mixture of their predictive densities. The Jensen Shannon Divergence between two predictive densities can be rewritten as the Mutual Information between this mixture of densities and a model classifier

$$\begin{aligned}
&D_{js}\left(Y|M_1, Y|M_2\right) \\
=&\sum_{m\in\{M_1,M_2\}} p(m) \int_Y p(y|M=m) \log \left(\frac{p(y|M=m)}{\sum_{m\in\{M_1,M_2\}} p(m)p(y|M=m)} \right) dy \\
=&\int_Y \sum_{m\in\{M_1,M_2\}} p(m)p(y|M=m) \log \left(\frac{p(y|M=m)}{\sum_{m\in\{M_1,M_2\}} p(m)p(y|M=m)} \right) dy \\
=&\int_Y \sum_{m\in\{M_1,M_2\}} p(y,m) \log \left(\frac{p(y,m)}{p(y)p(m)} \right) dy \\
=&I(Y,M)
\end{aligned} \tag{27}$$

Informally, measuring a quantity that maximizes this mutual information maximizes the reduction of uncertainty with respect to determining which of the two distributions the new measurement came from. This supports the JSD as a quantity useful for model selection.

S4. Analytical expressions for the linear models

To gain insight into the ability of the method at selecting optimal experiments, the methodology is applied to linear regression models. An advantage of using a model linear in the parameters is that analytic expressions for the Bayes factors are possible. Linear regression models are models of the form:

$$y(t) = \sum_{i=1}^{L} \theta_i B_i(t) + \epsilon \tag{28}$$

Where $\vec{\theta}$ represents a parameter vector and $\mathbf{B}$ constitutes a design matrix with basis functions $B_i(t)$. Given that σ is known and the prior distribution over the parameters is a Gaussian with standard deviation ξ, these models are linear in the parameters and the mean and covariance matrix of the posterior distribution is given by:

$$\mu = \left(\mathbf{B}^T\mathbf{B} + \frac{\sigma^2}{\xi^2}\mathbf{I}\right)^{-1} \mathbf{B}^T\mathbf{y} \tag{29}$$

$$\mathbf{\Sigma} = \sigma^2 \left(\mathbf{B}^T\mathbf{B} + \frac{\sigma^2}{\xi^2}\mathbf{I}\right)^{-1} \tag{30}$$

Furthermore, the marginal likelihood $p(y|M)$ used to compute the Bayes factor can be computed analytically as:

$$(2\pi)^{-\frac{m}{2}} |\mathbf{\Omega}|^{-\frac{1}{2}} e^{-\frac{1}{2}\mathbf{y}^T\mathbf{\Omega}^{-1}\mathbf{y}} \tag{31}$$

with:

$$\mathbf{\Omega} = \sigma^2\mathbf{I} + \xi^2\mathbf{B}\mathbf{B}^T \tag{32}$$

S5. Nonlinear model equations

Fluxes	Stoichiometry
$f_1 = k_1[A]$	$[\dot{A}] = -f_1 + f_2$
$f_2 = k_2[Ap]$	$[\dot{Ap}] = f_1 - f_2$
$f_3 = k_3 \frac{1}{k_9 + [BpCp]}[B][Ap]$	$[\dot{B}] = -f_3 + f_4$
$f_4 = k_4[Bp]$	$[\dot{Bp}] = f_3 - f_4 - f_5 + f_6$
$f_5 = k_5[Bp][Cp]$	$[\dot{C}] = -f_9 + f_{10}$
$f_6 = k_6[BpCp]$	$[\dot{Cp}] = -f_5 + f_6 - f_{10} + f_9$
$f_7 = k_7[D]$	$[\dot{BpCp}] = f_5 - f_6$
$f_8 = k_8[Dp]$	$[\dot{D}] = -f_7 + f_8$
$f_9 = k_{10}[C]$	$[\dot{Dp}] = f_7 - f_8$
$f_{10} = k_4[Cp]$	

Expressions for model one.

Fluxes	Stoichiometry
$f_1 = k_1 \frac{1}{k_9 + [BpCp]}[A]$	$[\dot{A}] = -f_1 + f_2$
$f_2 = k_2[Ap]$	$[\dot{Ap}] = f_1 - f_2$
$f_3 = k_3[B][Ap]$	$[\dot{B}] = -f_3 + f_4$
$f_4 = k_4[Bp]$	$[\dot{Bp}] = f_3 - f_4 - f_5 + f_6$
$f_5 = k_5[Bp][Cp]$	$[\dot{C}] = -f_9 + f_{10}$
$f_6 = k_6[BpCp]$	$[\dot{Cp}] = -f_5 + f_6 - f_{10} + f_9$
$f_7 = k_7[D]$	$[\dot{BpCp}] = f_5 - f_6$
$f_8 = k_8[Dp]$	$[\dot{D}] = -f_7 + f_8$
$f_9 = k_{10}[C]$	$[\dot{Dp}] = f_7 - f_8$
$f_{10} = k_4[Cp]$	

Expressions for model two.

Fluxes	Stoichiometry
$f_1 = k_1[A]$	$[\dot{A}] = -f_1 + f_2$
$f_2 = k_2[Ap]$	$[\dot{Ap}] = f_1 - f_2$
$f_3 = k_3 \frac{1}{k_9 + [Dp]}[B][Ap]$	$[\dot{B}] = -f_3 + f_4$
$f_4 = k_4[Bp]$	$[\dot{Bp}] = f_3 - f_4 - f_5 + f_6$
$f_5 = k_5[Bp][Cp]$	$[\dot{C}] = -f_9 + f_{10}$
$f_6 = k_6[BpCp]$	$[\dot{Cp}] = -f_5 + f_6 - f_{10} + f_9$
$f_7 = k_7[D]$	$[\dot{BpCp}] = f_5 - f_6$
$f_8 = k_8[Dp]$	$[\dot{D}] = -f_7 + f_8$
$f_9 = k_{10}[C]$	$[\dot{Dp}] = f_7 - f_8$
$f_{10} = k_4[Cp]$	

Expressions for model three.

Fluxes	Stoichiometry
$f_1 = k_1[A]$	$[\dot{A}] = -f_1 + f_2$
$f_2 = k_2[Ap]$	$[\dot{Ap}] = f_1 - f_2$
$f_3 = k_3 \frac{1}{k_9 + [D]}[B][Ap]$	$[\dot{B}] = -f_3 + f_4$
$f_4 = k_4[Bp]$	$[\dot{Bp}] = f_3 - f_4 - f_5 + f_6$
$f_5 = k_5[Bp][Cp]$	$[\dot{C}] = -f_9 + f_{10}$
$f_6 = k_6[BpCp]$	$[\dot{Cp}] = -f_5 + f_6 - f_{10} + f_9$
$f_7 = k_7[D]$	$[\dot{BpCp}] = f_5 - f_6$
$f_8 = k_8[Dp]$	$[\dot{D}] = -f_7 + f_8$
$f_9 = k_{10}[C]$	$[\dot{Dp}] = f_7 - f_8$
$f_{10} = k_4[Cp]$	

Expressions for model four.

S6. Spearman correlation JSD and Bayes factor

Consider linear models constructed from the following basis functions:

$$
\begin{aligned}
c_1(t) &= t && (33)\\
c_2(t) &= (.25t)^2 && (34)\\
c_3(t) &= (.1t)^3 && (35)\\
c_4(t) &= \sin(\alpha t^2) && (36)\\
c_5(t) &= e^{-|\beta t^2|} && (37)\\
c_6(t) &= \sin(\gamma t^3) && (38)\\
\alpha &\propto U[.1, .6] && (39)\\
\beta &\propto U[.05, .1] && (40)\\
\gamma &\propto U[.1, .6] && (41)
\end{aligned}
$$

Here $U[a, b]$ indicates a sample from a uniform distribution between a and b which is drawn upon basis function construction. Subsequently, regression models are assembled from these components by reordering these basis functions randomly. The reordered basis functions are referred to as C_i. The true model contains the first three of the reordered basis functions, while the different competing models are defined as follows:

$$
\begin{aligned}
M_1(t) &= \theta_1 C_1(t) && (42)\\
M_2(t) &= \theta_1 C_1(t) + \theta_2 C_2(t) && (43)\\
M_3(t) &= \theta_1 C_1(t) + \theta_2 C_2(t) + \theta_3 C_3(t) && (44)\\
M_4(t) &= \theta_1 C_1(t) + \theta_2 C_2(t) + \theta_3 C_3(t) + \theta_4 C_4(t) && (45)\\
M_5(t) &= \theta_1 C_1(t) + \theta_2 C_2(t) + \theta_3 C_5(t) && (46)\\
M_6(t) &= \theta_1 C_1(t) + \theta_2 C_3(t) + \theta_3 C_5(t) && (47)
\end{aligned}
$$

Note that model four is an over-parameterized version of the data generating model, while the last two models are wrong. Subsequently, using posterior samples corresponding to the true model, a distribution of post measurement Bayes factor is computed. Here the new measurement is computed as a posterior sample plus measurement noise. Subsequently, the mean of these Bayes factor is computed for every potential measurement, providing an expected Bayes factor. For these same predictions, Jensen-Shannon divergences are computed. As data prior to the design, data at time points $t_{old} = [-1, -.8, \ldots, .8, 1]$ was used. While potential experiments at $t_{new} = [-2.5, -2.3, \ldots, 2.3, 2.5]$ were considered. The true parameters were drawn from a normal distribution with a standard deviation of 2. The standard deviation of the noise was varied between .1 and 3.1, while the standard deviation of the noise on the new measurement was set to .3. The prior distribution on the parameters had width 10. The expected Bayes factor was based on 100 samples from the posterior distribution. Computing

the Spearman correlation coefficient (a measure of monotonicity) between the expected Bayes factor and JSD for 75 randomly generated models results in the following average correlation coefficients:

Test	Comparison	Spearman CC
Underparameterization	M_3 over M_1	$.90 \pm .22$
Underparameterization	M_3 over M_2	$.93 \pm .12$
Overparameterization	M_3 over M_4	$.88 \pm .11$
Misspecification	M_3 over M_5	$.91 \pm .10$
Misspecification	M_3 over M_6	$.94 \pm .04$

Higher dimensional models

Additionally, we have considered GLMs with larger numbers of parameters. The construction of the different models was performed analogously to the previous section, but using different basis functions. In this case, we used models comprised of damped oscillations.

$$c_1(t) = \exp(-.2\alpha t) \tag{48}$$
$$c_2(t) = \exp\left(-.2\alpha t^2\right) \tag{49}$$
$$c_3(t) = \exp\left(-.2\alpha t^3\right) \tag{50}$$
$$c_4(t) = \exp(-.2\alpha t)\sin(\alpha\pi t) \tag{51}$$
$$c_5(t) = \exp(-.2\alpha t)\cos(\alpha\pi t) \tag{52}$$
$$\tag{53}$$

Models are constructed using random combinations of these basis functions using integer values for α while ensuring every combination of alpha and basis function is only included once. The true model consists of 24 basis functions. The alternatives are defined analogously to the previous section. Relationship between the expected Bayes factor and Jensen Shannon Divergence is probed using a ranked correlation coefficient. Results corresponding to a JSD lower than 0.01 were filtered out. As data prior to the design, data at time points $t_{old} = [0, .01, \ldots, 1]$ and $t = 1.5$ and 1.75 was used. Each iteration true parameters were drawn from a uniform distribution. The standard deviation of the noise was set to 5, while the standard deviation of the noise on the new measurement was set to 1. The prior distribution on the parameters had width 25. A typical result is depicted in Figure 1. Results (based on 15 random models) are as follows:

Test	Comparison	Spearman CC
Underparameterization	M_3 over M_1	$.97 \pm .02$
Underparameterization	M_3 over M_2	$.98 \pm .02$
Overparameterization	M_3 over M_4	$.91 \pm .09$
Misspecification	M_3 over M_5	$.97 \pm .02$
Misspecification	M_3 over M_6	$.93 \pm .09$

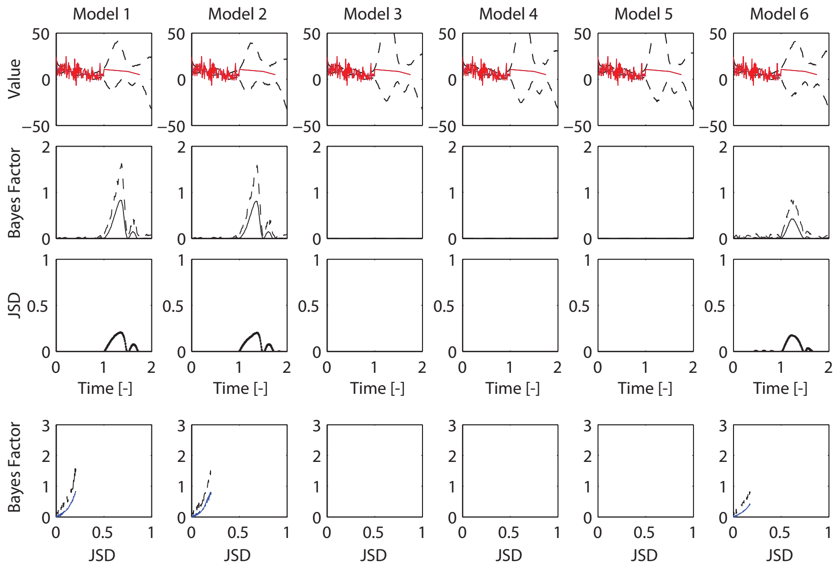

Figure 1: Comparing the Jensen Shannon Divergence to the updated Bayes factor. Inference was performed based on data generated from the true model. Bayes factor updates pointing to the correct model were calculated given a new measurement which was based on the inferred posterior predictive distribution of the true model. Top row: Predictive distribution of of the different models shown in dashed lines. Data shown in red. Second row: Predictive distribution of Bayes factors between model indicated by the column and the true model (third column). Third row: Jensen Shannon divergence between the relevant Posterior Predictive Distributions. Bottom row: Relation between the updated Bayes factors and the JSD. Note that the relation is nearly monotonic indicating its usefulness as a predictor of experimental efficacy.

S7. Sampling bigger design matrices

When designing multiple experiments, each additional experiment constitutes an additional direction in experimental design space. For the 2D examples in the main text filling this matrix entirely was not a problem. When designing a larger number of experiments sampling this entire space rapidly becomes infeasible due to the curse of dimensionality. This is when one must resort to sampling methods. As a proof of principle, we present a sampler based on ideas from Sequential Importance Resampling to sample these spaces. The sampler works by executing a number of steps

- 1. Sample Q particles from a uniform distribution over the design space. When the number of samples is reasonably small, one can use Latin Hypercube sampling to improve the coverage of the design space.
- 2. Evaluate JS divergences for each of the samples and clip any negative values for the JS to zero.
- 3. Compute cumulative distribution function CDF_{js} for the JS divergence and normalize to a maximum of 1.
- 4. Generate νQ samples from a uniform distribution X_i $U[0,1]$. Generate particles for next iteration by using values corresponding to particles from the old set where $X_i > CDF_{js}$ for the first time. Generate $(1-\nu)\,Q$ particles from a uniform distribution over the design space.
- 5. Perturb particles using a perturbation kernel until none of the particles correspond to samples that were already evaluated.
- 6. Goto 2 until desired number of iterations has been reached.

This algorithm is straightforward to implement and results in regions with high values for the JSD to be explored more densely. Optionally, one can factor in an experimental cost in the sampling criterion by multiplying the associated JSD before computing the CDF. Note how in this implementation every iteration involves a small fraction of the particles (ν) being sampled from the prior. This is to avoid missing specific high JSD regions in parameter space in longer runs. It allows the algorithm to find new regions of high JSD once the initial ones are densely sampled. An additional advantage is that this approach allows real time monitoring of intermediate results from the experimental matrix.

The sampling was compared to a full determination of the design matrix in the 2D case. The comparison was performed by determining the maximum JSD found during the sampling. Secondly, by determining the fraction of JSD accounted for when comparing to the full sampling procedure. This fraction is computed by dividing the sum of the JSD for the sampled points by the sum over the JSD values for the entire matrix. These two quantities are displayed in Figure 2 for $Q = 100$ particles over 100 iterations.

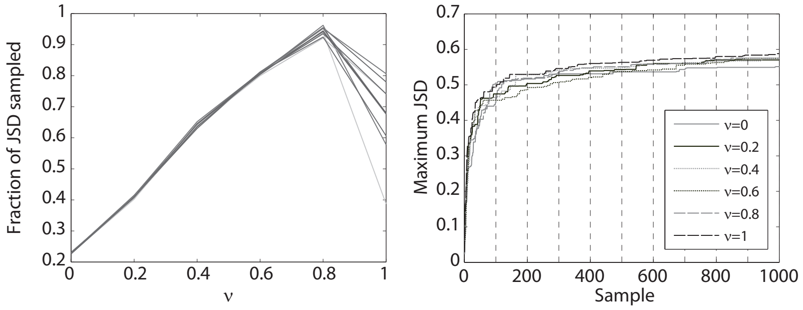

Figure 2: Sampling of the experiment design matrix in the 2D case. Left: Fraction of total JSD found versus various values for ν. Shown are ten independent samplings. Right: Maximum JSD found up to a certain iteration for different values of ν.

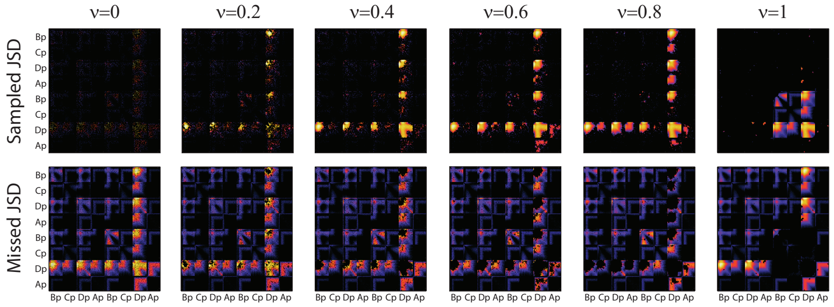

Figure 3: Sampling of Jensen-Shannon divergences for various combinations of observables. Shown are the results for 100 iterations using 100 particles. This is 12% of the evaluations required to sample the entire matrix. Top: JSD sampled using the importance resampling approach. Bottom: Regions not sampled.

Note how the maximum value found for the JSD does not seem to be very sensitive to the value chosen for ν, while the fraction is strongly affected. For most sampling runs, high values for ν turned out to be better, though the variability between different runs increases drastically for $\nu = 1$. The actual design matrices corresponding to the different values for ν are shown in Figure 2. Note how the high JSD regions are sampled more densely for higher ν, yet for $\nu = 1$, some regions are completely missed.

Despite that the efficiency of the sampling likely depends on the posterior predictive distributions (the relative size of the dense regions) and the dimensionality of the problem, the improvement over simple random sampling $\nu = 0$ is likely to remain.

S8. Choosing the size parameter for the density estimation

Computation of the Jensen Shannon Divergence requires estimating predictve densities. In this work we propose using a kNN estimator to perform this density estimation. To use kNN density estimation, we need to choose k, which refers to the number of nearest neighbours that the estimated volume has to enclose. Shown in Figure 4 are the OED matrices based on different values for k. The patterns observed in the matrices are well conserved in the sense that one would come to the same conclusion regarding which experiments are useful.

To test how well density estimation works in higher dimensions some tests were performed using multivariate Gaussian distributions. Initially, we estimated JSD between multivariate distributions with the same standard deviation for every variable. The correlation between the first and second variable was varied between 0 and 1. This correlation was set positive for model 1, and negative for model 2. The other correlations were set to zero. The results are shown in Figure 5. What can be seen is that density estimation using the kNN estimator is more insensitive to the actual estimator parameter k. What can also be seen is that the Parzen estimate quickly degrades for higher dimensions.

When the dispersion of the different scales is very different, relations between the different predictions can become hard to detect however. See Figure 6 to see an example where the different dimensions had standard deviations given by $10^{\frac{1}{2}q}$ with q given by $[1, 2..., 10]$. What can be seen is that the additional variables operating at larger scales drown out the different correlations present in the distributions. What can be seen however is that the kNN is more robust to these additional nuisance dimensions in the sense that the relation between the JSD and correlation coefficient is more apparent. The case where the correlation coefficients are set to the same value is shown in Figure 7. Summarizing, density estimation in low dimensional spaces is usually not too problematic. For higher dimensional spaces problems can occur however. Particularly when the different predictions are on different scales. We recommend initially designing only a few experiments simultaneously and incrementally adding more experiments. If widely disparate scales are present in the PPDs, one could opt for prescaling the different predictions or log-transforming the different quantities before performing the density estimation.

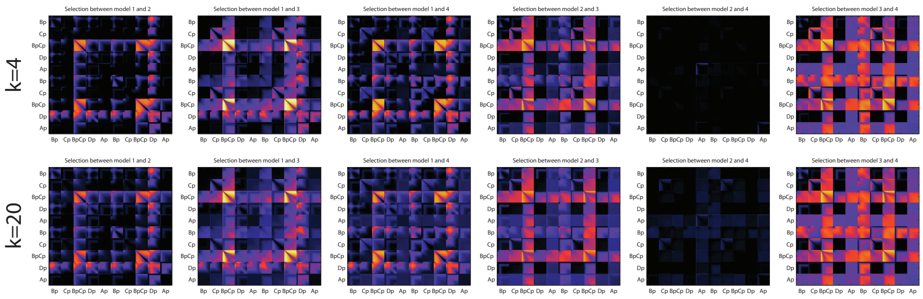

Figure 4: Jensen Shannon divergences for each comparison of the non-linear models in the main paper. Each axis represents a single measurement. Each tile corresponds to a combination of two state variables where the space within each tile corresponds to the actual time point at which the state variable is measured. The first five predictions correspond to the same experimental condition as during the original inference ($u = 1$) while the second five predictions correspond to a different stimulus ($u = 2$). The circles denote the different experiments that were performed. Top row was determined using $k = 4$, bottom row using $k = 20$. The location of regions with large JSD are well conserved.

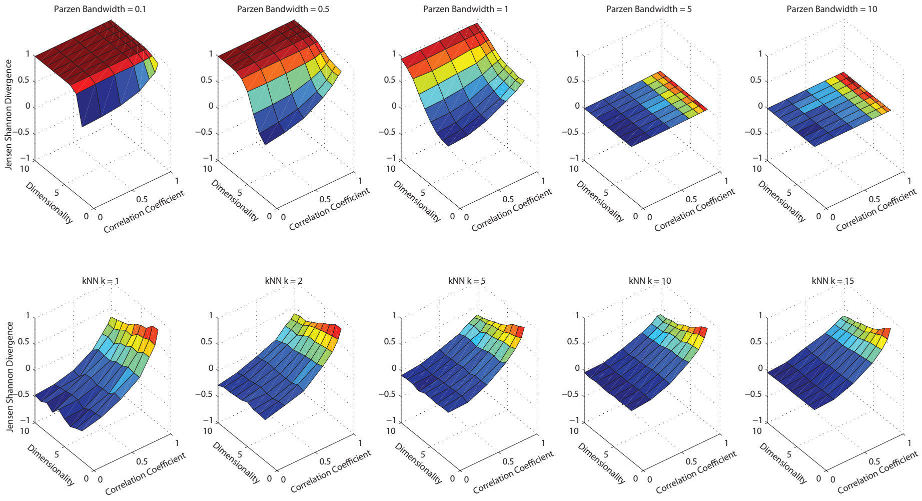

Figure 5: Jensen Shannon divergences between two multivariate normal distributions with varying number of dimensions. Correlation between first and second variable specified as denoted by the x-axis. Correlation for distribution one is positive, while the correlation for the second distribution is negative. Other correlations set to zero. Standard deviations set to the same value. JSD in the top row was estimated using KDE. JSD in bottom row using kNN.

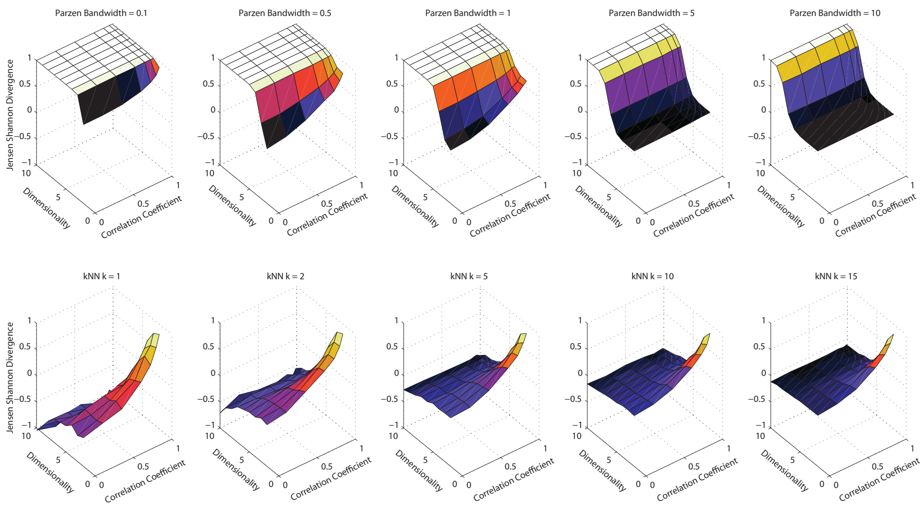

Figure 6: Jensen Shannon divergences for multivariate normal distributions with varying number of dimensions. Correlation between first and second variable specified as denoted by the x-axis. Correlation for distribution one is positive, while the correlation for the second distribution is negative. Other correlations set to zero. Standard deviations vary exponentially. JSD in the top row was estimated using KDE. JSD in bottom row using kNN.

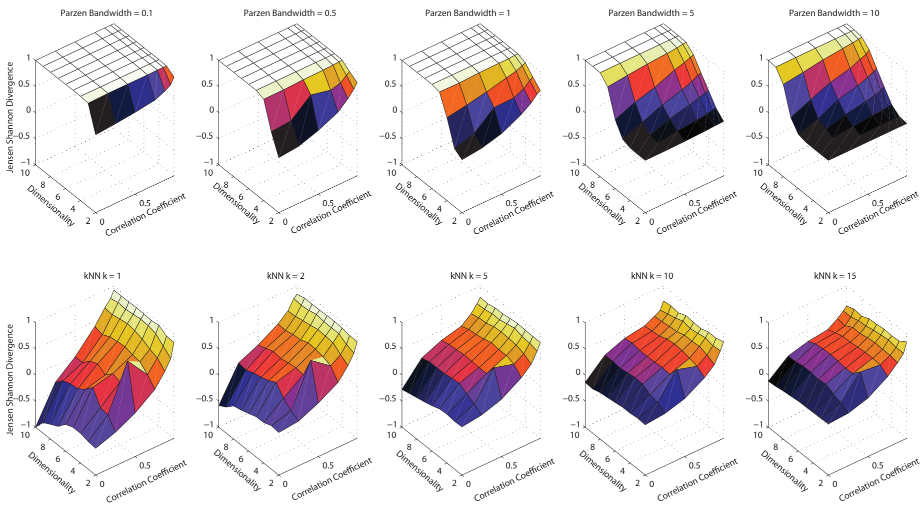

Figure 7: Jensen Shannon divergences for multivariate normal distributions with varying number of dimensions. Correlation between first and second variable specified as denoted by the x-axis. Correlation for distribution one is positive, while the correlation for the second distribution is negative. JSD in the top row was estimated using KDE. JSD in bottom row using kNN.

References

[1] B. Calderhead and M. Girolami. Estimating bayes factors via thermodynamic integration and population mcmc. *Computational Statistics & Data Analysis*, 53(12):4028–4045, 2009.

[2] C.J. Geyer. Practical markov chain monte carlo. *Statistical Science*, pages 473–483, 1992.

[3] Ryan N Gutenkunst, Joshua J Waterfall, Fergal P Casey, Kevin S Brown, Christopher R Myers, and James P Sethna. Universally sloppy parameter sensitivities in systems biology models. *PLoS Comput Biol*, 3(10):e189, 10 2007.
